# Characterizing the Impact of Commercial Pollen Substitute Diets on the Level of *Nosema* spp. in Honey Bees (*Apis mellifera* L.)

**DOI:** 10.1371/journal.pone.0132014

**Published:** 2015-07-30

**Authors:** James C. Fleming, Daniel R. Schmehl, James D. Ellis

**Affiliations:** Entomology and Nematology Department, 970 Natural Area Drive, University of Florida, Gainesville, Florida, 32611, United States of America; San Diego, UNITED STATES

## Abstract

Western honey bee (*Apis mellifera* L.) populations face declines commonly attributed to pesticide, pathogen, and parasite stress. One way beekeepers combat these stressors is by providing supplemental protein diets to honey bee colonies to ensure adequate colony nutrition. However *Nosema* spp., a microsporidian parasite of the honey bee, is thought to be associated closely with a colony’s nutritional intake, thus possibly negating any benefit the bees otherwise would have received from a nutritional supplement. Through three objectives, we examined how adult bees’ consumption of wildflower pollen or commercial pollen substitute diets affected *Nosema* levels in the bees’ midguts. For our first objective, we investigated how method of inoculation with *Nosema* affects infection levels in inoculated bees. Bees were infected with spores of *Nosema* four days after emergence. On day 15, bees were collected from the cages and *Nosema* spores were quantified. We found that inoculation through the pollen diet resulted in the highest *Nosema* levels in inoculated bees. In our second and third objectives, we provided the test diets to caged, newly emerged bees for a period of 15 days. Bees consuming pollen and a sucrose solution had more *Nosema* in their midguts than did bees consuming the sucrose solution alone (control). The overall volume of diet consumed by the bees did not correlate with the level of *Nosema* in their midguts. The level of *Nosema* was higher in bees fed certain commercial pollen substitute diets than in bees fed wildflower pollen. Our study illustrates how providing nutritional supplements to adult honey bees can impact the intensity of *Nosema* in their midguts.

## Introduction

The Western honey bee, *Apis mellifera* (Linnaeus, 1758), provides crucial pollination services for much of the world’s crops [1,2]. In the United States, the value of crops dependent on honey bees is in excess of $14.6 billion [3]. Honey bee colony populations have declined at a high annual rate over the last decade [4]. The exact cause for this decline is unknown and has been attributed to pesticide exposure, inadequate nutrition, parasites, pathogens, climate change, management practices, and other stressors [5–17].

Good colony nutrition, such as adequate protein and carbohydrate stores, is believed to help bees to resist or tolerate many of the stressors associated with modern apiculture [18–20]. Honey bees are highly dependent on foodstuffs stored within the hive. Worker bees do not have substantial protein reserves in their bodies; therefore, they require a daily diet of about 3.4–4.3 mg of pollen, depending upon their age, to make up this nutritional deficiency. A typical 10-frame colony consumes between 13.4 and 17.8 kg of pollen annually [21]. Nearly all protein and vitamins needed by bees are derived from pollen stored as bee bread inside the hive when pollen otherwise is not available in the environment [18,22].

Beekeepers in the U.S. routinely feed colonies pollen substitute diets when they believe bees are experiencing a nutrition dearth or if the incoming resources are believed to be of low or insufficient quality. For example, substitute pollen diets, which commonly consist of a protein source derived from soy, wheat, or lentils and is fortified with essential vitamins, often are fed to migratory colonies when the colonies are being used to pollinate crops. These diets help serve as a vital protein source to colonies [23] and offset the poor nutritional conditions frequently associated with agricultural landscapes [24,25]. Feeding colonies pollen substitutes increases annual honey yield, brood health, worker longevity, and worker development [22]. Though the primary protein source utilized by colonies is pollen [18], beekeepers feed colonies pollen substitutes because the diets are more widely available and affordable than is natural, bee-collected pollen [26].

Colonies with inadequate nutrition have a higher risk of experiencing negative effects associated with other stressors in the colony, such as pesticide exposure [27–29] and pathogen infection. Regarding the latter, *Nosema* spp *(Nosema)* is a pathogen of particular interest because of its close association with the honey bee midgut and bee nutrient adsorption. *Nosema* spp. are obligate intracellular fungal parasites of insects [30,31] and they have a worldwide distribution [32,33]. *Nosema apis* [34] and *Nosema ceranae* are two species that commonly affect the western honey bee. *Nosema* attacks the epithelial lining of the bee’s midgut. There, the pathogen multiplies and is spread throughout the colony via normal bee trophallaxis and uncontrolled defecation [22,35]. A *Nosema* infection is most problematic to colony health in the winter and early spring [36]. *Nosema* infection prevents adequate nutrient digestion and absorption in a bee’s midgut [37] contributing to an increase in appetite and a reduction in activity [20].

Beekeepers feed pollen substitutes to colonies to increase colony strength and reduce colony susceptibility to pathogens, such as *Nosema*. This practice may, however, be counterproductive given that *Nosema* competes with bees for nutrition, possibly leading to increased *Nosema* levels in bees provided pollen substitute diets. Thus, we hypothesized that *Nosema* infections may be worse in bees that have fed on natural pollen or beekeeper-provided pollen substitute diets than in bees that have not. To better explore the influence of diet on *Nosema* infections in bees, we first determined if the method of inoculating bees with *Nosema* affects overall *Nosema* levels in the inoculated bees. While there are standardized methods for *Nosema* inoculation [38], it is not known how the inoculation medium (i.e. sucrose solution or pollen) influences *Nosema* pathogenicity. Second, we determined the contribution of the amount of pollen consumed by a bee to the level of *Nosema* infection it had. Porrini et al. [39] demonstrated that bees fed pollen had higher *Nosema* levels than those fed less nutritious diets consisting of high fructose corn syrup and soy derived protein. Consequently, we hypothesized that *Nosema* levels in bees would correlate with increased pollen consumption and increase linearly relative to the amount of pollen the bees ingested. Finally, we determined the contribution of the consumption of pollen substitute diets by bees to *Nosema* levels in the bees during the fall and spring seasons. We hypothesized that bee consumption of diets with differing nutritional content would result in different *Nosema* spore numbers in the bees since Porrini et al. [39] showed *Nosema* levels varied between bees fed pollen and bees fed high fructose corn syrup containing a commercial mixture of amino acids and vitamins. By measuring the contribution of diet to the number of *Nosema* spores in a bee, we can guide beekeepers in choosing the most suitable pollen substitute diet for improving their colony health.

## Materials and Methods

### Obtaining honey bees

European-derived honey bees were obtained from the Bee Biology Research Unit at the University of Florida (29.627042N,-82.356373W) during fall 2013 and spring 2014. Frames of capped brood from multiple hives were collected and maintained in a reach-in incubator (Percival 136VS) at 34.5°C and 65% relative humidity (RH). After a period of 24 hours, newly emerged workers were pooled from the collected frames and 15 adult bees were placed into each bioassay cage.

### Bioassay cages

Bioassay cages (**S1 Fig Bioassay cage with adult honey bees**), modified from Williams et al. [40], were constructed of a clear 295 mL cup (Amscan Big Party Pack, Simply Unforgettable Party Shop). The cup was inverted so that the bottom of the cup faced upwards. Two feeder holes were inserted in the plastic cup using a brass cork borer (size 7, 12.5 mm in outside diameter) that was heated over a Bunsen burner. One of the holes was inserted in the top of the cage (bottom of the cup) to accommodate a feeder containing 50% sucrose solution (1:1 sucrose: water, w/v) while the other hole was inserted on the side to accommodate a water feeder. The feeders were constructed of 1.5 ml Eppendorf centrifuge tubes with two holes (1.2 mm) drilled into the tubes for the bees to acquire the respective solution. One circular ventilation hole (2.3 cm) was inserted through the side of each cage using a heated cork borer. A piece of charcoal-colored, fiberglass screen, size #5 (Phifer, Lowes), was affixed over the hole using a hot glue gun. The top portion of a 100 mm × 15 mm culture plate (Fisherbrand) was placed under the bottom of the cage and secured to the cage using a rubber band placed longitudinally around the cage. A hole was inserted (using a heated, size 7 cork borer) into the center of the culture plate to accommodate base mount queen-rearing cups (JZ_BZ, Mann Lake LTD.) that were used to deliver the pollen or pollen substitute diet to the bees. A 6.5 cm × 5 cm piece of wax foundation (Walter T. Kelley Co.) was placed on the inside of the cage and secured using five, 1.27 cm long brass fasteners (Walmart). Five holes were inserted into the side of the plastic cup using a soldering iron to accommodate the brass fasteners used to secure the wax to the side of the cage.

### *Nosema* quantification

*Nosema* spores for inoculation were collected by homogenizing, purifying, and quantifying spores as described in Fries et al. [38]. Forager bees were collected at the entrance of a hive infected with *Nosema*. The bee’s abdomens were removed and homogenized in 2 mL deionized (DI) H_2_O using a mortar and pestle during the fall studies and a FastPrep-24 with TeenPrep Adapter (MP Biomedicals) during the studies undertaken in spring. Ten mL of DI H_2_O was added to the homogenized tissue prior to filtering the homogenate. The homogenate was filtered through a two-step gravity filter with the first funnel lined with charcoal fiberglass screen, size #5 (Phifer, Lowe’s), and the second filter lined with sheer drapery (Batiste, JoAnn’s Fabric). The supernatant was centrifuged (Eppendorf 5810R) at 3000 RPM for 5 minutes. The supernatant was discarded and the resulting pellet was reconstituted in 10 mL DI H_2_O before being centrifuged a second time at 3000 RPM for 5 minutes to eliminate fat body fragments and other debris. After an additional round of reconstituting and centrifuging as above, the pellet was reconstituted in 5 mL DI H_2_O, thus creating an inoculation stock. To quantify the inoculum, 10 μL of inoculum was diluted with 90 μL DI H_2_O. 10 μL of the dilution was pipetted onto a hemocytometer (INCYTO C-CHIP). Spores were counted within the 25 squares in the innermost grid at 400× magnification using a phase contrast microscope (Leica).

### The impact of inoculation route on *Nosema* levels in bees

A cage study was established to determine whether inoculation route and pollen intake impacts *Nosema* levels in bees. Cages were established with 15 newly emerged bees (<24 hours old) and received one of four treatments with five replications of each treatment. Bees in cages were fed (1) sucrose solution only with no *Nosema* inoculum (control), (2) sucrose solution only with *Nosema* inoculum in the sucrose solution, (3) sucrose solution and pollen with *Nosema* inoculum in the sucrose solution, or (4) sucrose solution and pollen with *Nosema* inoculum in the pollen. The *Nosema* inoculum was added directly to the “pollen mixed with 50% w/v sucrose solution” (denoted as “pollen” hereafter) or 50% sucrose solution and fed to the bees at a concentration of 200,000 spores per bee when bees were four days old. When appropriate, the inoculum was delivered through 0.15 g of wildflower pollen provided to bees in each cage (pollen inoculum) or through 125 μL of 50% sucrose solution (w/v) provided to bees in each cage (sucrose inoculum). For each cage, the inoculum, whether delivered through pollen or sucrose, was replaced after a period of 24 hours with fresh diet containing no spores. Bees in treatments receiving pollen were given 0.4 g wildflower pollen every four days. Bees in all cages were provided with 50% (w/v) sucrose *ad libitum*. Bioassay cages were kept in an incubator (Binder model # BF-400-UL) at 34.5°C and 40% RH. The humidity was maintained using a saturated sodium chloride solution. Dead bees were removed daily.

All living bees were collected at 15 days old and anesthetized by placing the cages in a -20°C freezer. Once the bees were anesthetized, abdomens were collected from 10 bees per cage and homogenized in 2 mL deionized (DI) H_2_O using a mortar and pestle. *Nosema* levels were quantified. We conducted a one-way analysis of variance (ANOVA) recognizing treatment (method of inoculation) as the independent variable and spore count as the dependent variable to determine whether there were differences in the *Nosema* spores between the treatment groups (JMP 10, SAS, Cary, NC).

### The impact of pollen consumption on *Nosema* levels in bees

Honey bees were fed different amounts of wildflower pollen over a 15 day period to determine the contribution of pollen consumption to their *Nosema* levels. Cages were established with 15 newly emerged bees (<24 hours old) and grouped into one of six treatments with five cages per treatment. The caged bees were provided irradiated (to eliminate any potential pesticide contaminants) wildflower pollen (acquired from Straughn Farms, Waldo, Florida) in different quantities of 0 g (control), 0.1 g, 0.2 g, 0.3 g, 0.4 g, and 0.5 g of pollen per cage every four days such that the treatment groups received either 0 g, 0.4 g, 0.8 g, 1.2 g, 1.6 g, and 2.0 g of pollen per cage over the course of the study, according to treatment group designation. The caged bees were inoculated on day four with a concentration of 100,000 spores per bee delivered through 125 μL of 50% sucrose solution (w/v). Once the inoculated sucrose solution was consumed, the bees received 50% sucrose solution *ad libitum* throughout the remainder of the experiment while the pollen in the cages was replaced every four days. Dead bees were removed daily and the associated mortality was recorded. At the end of 15 days, the bees were anesthetized at -20°C. Two pooled samples of five abdomens per sample were homogenized separately from each cage. *Nosema* spores were purified and quantified. The two samples from each cage were averaged together to analyze the spores per bee from each cage. Treatment differences in *Nosema* levels, bee mortality, and diet consumption data were determined using a one way ANOVA recognizing treatment (amount of pollen provided) as the independent variable and *Nosema* level (spores/bee), bee mortality and diet consumption as dependent variables (JMP 10, SAS, Cary, NC).

### The impact of bee consumption of commercial diets on *Nosema* levels

We established cages with 15 newly emerged workers (<24 hours old) to measure the impacts of commercial pollen substitute diets on *Nosema* spore levels in bees. Bees in ten cages per treatment, for a total of six treatments, were fed for a period of 15 days after establishment. Treatment groups consisted of bees fed sucrose solution and one of six possible diet regimens: (1) no pollen diet provided (control), (2) irradiated wildflower pollen (acquired from Straughn Farms, Waldo, Florida), (3) Ultra Bee powder containing 60% crude protein (Mann Lake), (4) Bee-Pro powder containing 48.5% crude protein and 3.8% crude fat (Mann Lake), (5) MegaBee powder containing 40% protein and 4% fat (Dadant), or (6) MegaBee Winter Patty containing 3% protein with carbohydrates and Honey Bee Healthy additive (Dadant). Each diet had the same amount of sucrose solution, but differing amounts of water to ensure a similar consistency across the diets. The diets were mixed according to the following ratios: 50:50 wildflower pollen (3 g pollen with 3 mL 50% sucrose solution, 1:2.5 Ultra Bee (3 g product powder with 3 mL 50% sucrose solution and 4.5 mL DI H_2_O), 1:2.5 Bee-Pro (3 g product powder with 3 mL 50% sucrose solution and 4.5 mL DI H_2_O), 1:2.5 MegaBee powder (3 g product powder with 3 mL 50% sucrose solution and 4.5 mL DI H_2_O), and 3:1 MegaBee Winter Patty (3 g patty with 1 mL 50% sucrose solution to increase malleability). 0.4 g of the respective diet was provided to bees in each cage and replaced every four days. The weight of the diet, indicative of diet consumption, was determined after it was removed for the cage. All cages were provided with 50% (w/v) sucrose *ad libitum*. Dead bees were removed daily. Bee mortality and diet consumption were recorded throughout the course of the experiment. Three days after cage establishment, bees were fed a *Nosema* inoculum at a concentration of 200,000 spores per bee in a 50% (w/v) sucrose solution. At the end of 15 days, the bees were anesthetized in a -20° C freezer. Two pooled samples of five abdomens per sample were homogenized separately from each cage. *Nosema* spores were purified and quantified. The two samples from each cage were averaged together to analyze the spores per bee for each cage. Differences in *Nosema* levels, bee mortality, and diet consumption (dependent variables) data were determined using a one-way ANOVA recognizing treatment (diet type) as the independent variable (JMP 10, SAS, Cary, NC).

This study was repeated in two seasons (study 1- fall 2013, study 2- spring 2014) to characterize any possible seasonal variations in diet impacts on *Nosema* levels in bees. The same pollen substitute diets were evaluated during both seasons. However, in spring 2014, the uninoculated wildflower pollen treatment was omitted and replaced with two other treatment groups fed only sucrose. One of the sucrose treatment groups received the *Nosema* inoculum in the sucrose, while the other sucrose treatment received no *Nosema* inoculum.

To confirm that *Nosema* was not present in the commercial diets prior to inoculation, caged bees were fed one of six treatments (sucrose only, wildflower pollen, Ultra Bee, Bee-Pro, MegaBee, MegaBee Winter Patty). The bees did not receive any *Nosema* inoculation. Five cages of 15 newly emerged (<24 hours old) bees per cage were established for each treatment group for a period of 15 days. Caged bees were fed 50% sucrose *ad libitum* and 0.4 g of the appropriate diet was placed in the respective treatment cages. Every four days, diets were replaced and bee mortality was recorded throughout the course of the experiment. At the end of 15 days, the bees were anesthetized in a -20° C freezer. Two pooled samples of five abdomens per sample were homogenized separately from each cage. *Nosema* spores were purified and quantified as previously described. The two samples from each cage were averaged together to determine the spores per bee from each cage. Differences in *Nosema* spore levels, bee mortality, and diet consumption (dependent variables) data were analyzed using a one-way ANOVA recognizing treatment (diet type) as the independent variable (JMP 10, SAS, Cary, NC).

## Results

### The impact of inoculation route on *Nosema* levels in bees

**Fig 1:**
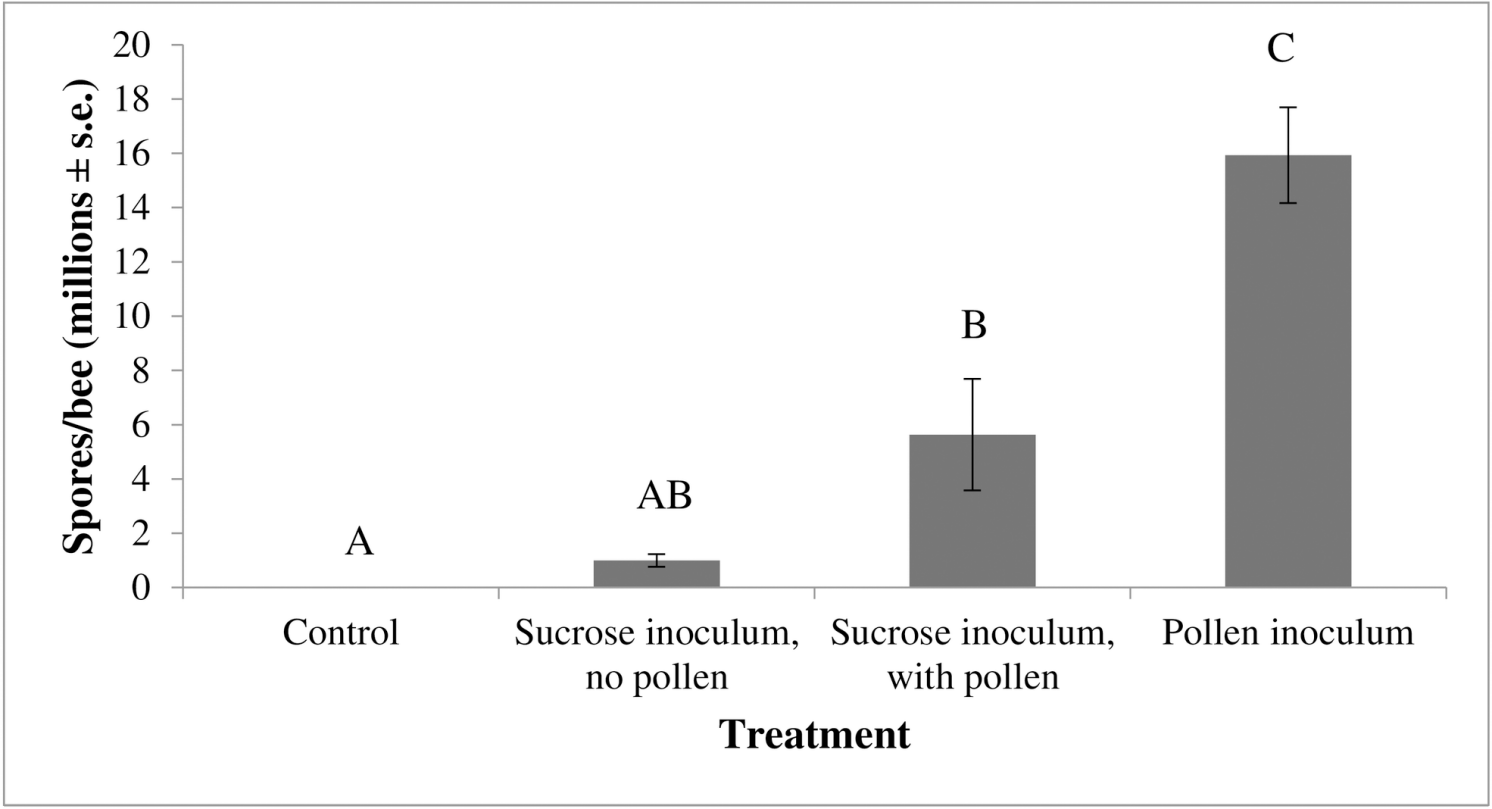
The impact of inoculation method on *Nosema* levels in bees. Data are the average number of spores per bee (in millions) with the error bars denoting standard error. N = 17 for all treatment groups. The treatment groups are (1) control (bees fed a sucrose solution, no inoculum) (2) sucrose inoculum, no pollen (bees inoculated with *Nosema* through a sucrose solution and given no pollen), (3) sucrose inoculum, with pollen (bees inoculated with *Nosema* through a sucrose solution and given pollen), and (4) pollen inoculum, with sucrose solution (bees inoculated with *Nosema* through pollen). Treatment (method of inoculation) significantly affected *Nosema* levels in bees (ANOVA, F_3,13_ = 16.7, p<0.0001). Posthoc Tukey-HSD pairwise comparisons identified significant differences between treatments (data with the same letter are not different at α ≤ 0.05).

Bees receiving the inoculum in the pollen had significantly elevated spore levels (15.9 million spores ± 1.8 million spores (n = 4), average number spores ± s.e. (n)) than those receiving the inoculum in the sucrose solution (5.6 million ± 2 million (n = 5), ANOVA, F_3,12_ = 16.7, p<0.0001, Fig 1). The *Nosema* levels in sucrose-inoculated bees fed pollen was higher than in sucrose-inoculated bees not fed pollen (1 million ± 0.23 million (n = 3)). There was a significantly greater number of *Nosema* spores in bees inoculated through the pollen diet than in those inoculated through the sucrose solution (Fig 1). However, a sucrose delivery of *Nosema* was determined to be preferable to *Nosema* delivery through pollen because all sucrose-inoculated cages received a consistent application of sucrose that the bees consumed completely. The consumption of pollen by the bees varied between cages, thus leading to fears of unequal inoculation rates among bees in cages receiving the inoculum through pollen.

### The impact of pollen consumption on levels in bees

**Fig 2:**
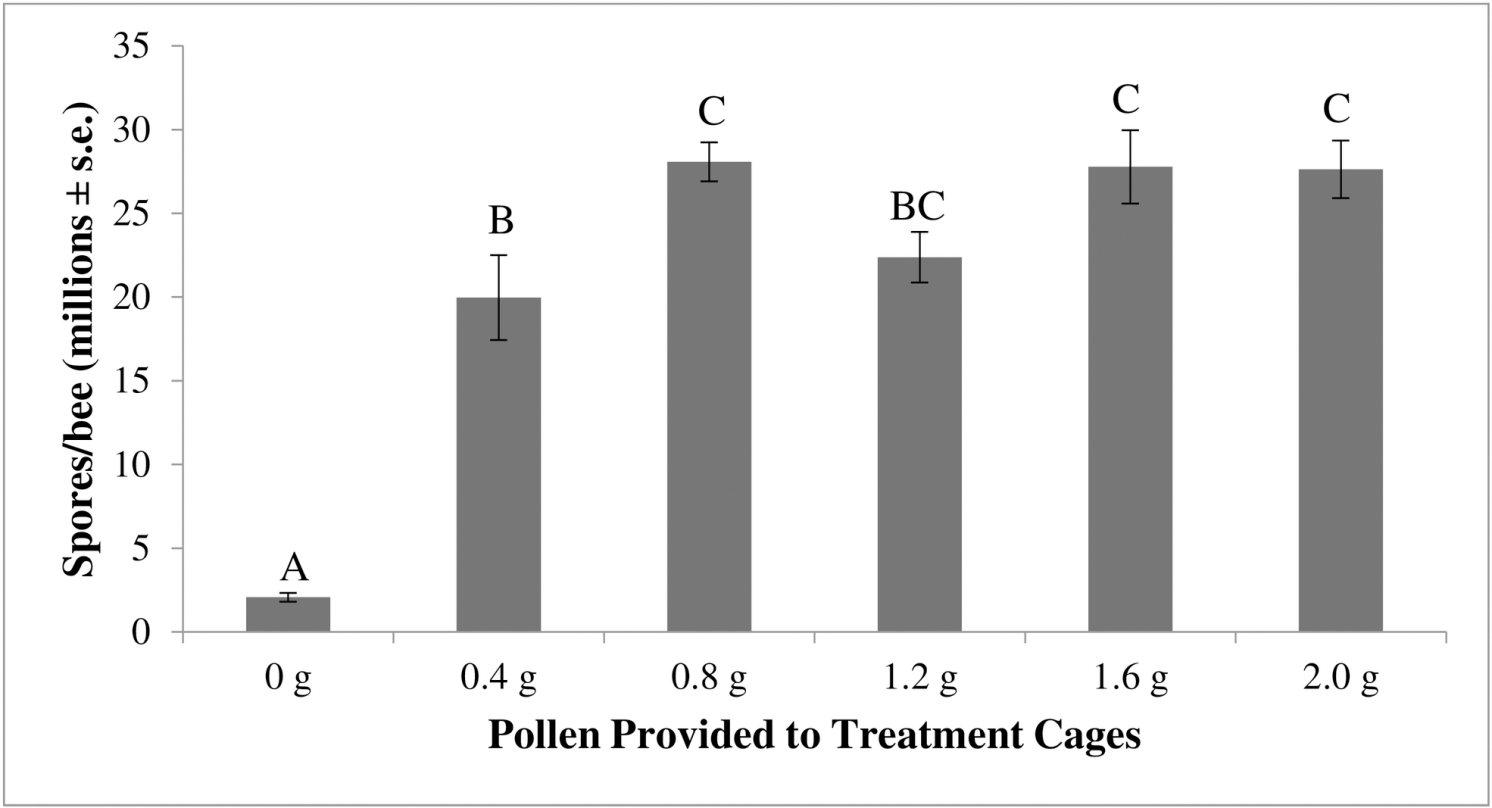
The effect of pollen consumption on *Nosema* levels in bees. Data are the average number of spores per bee (in millions) with the error bars denoting standard error. N = 30 for all treatment groups. The treatment groups (x axis) represent the amount of pollen given to bees/cage. Treatment (amount of pollen provided to the bees) significantly affected the spore levels in bees (ANOVA F_5,24_ = 33.6, p<0.0001). Posthoc Tukey-HSD pairwise comparisons identified significance between treatments (data with the same letter are not different at α ≤ 0.05).

The amount of pollen provided to the bees had a significant effect on *Nosema* levels (average number of spores ± s.e. (n = 30)) in the bees (ANOVA F_5,24_ = 33.6, p<0.0001, Fig 2). There were significantly more *Nosema* spores in bees fed 0.8 g (28.1 million spores/bee ± 1.0 million spores (n = 5)), 1.6 g (27.8 million spores/bee ± 1.9 million (n = 5)), and 2.0 g (27.6 million spores/bee ± 1.5 million (n = 5)) of pollen than bees fed only 0.4 g of pollen (20.0 million spores/bee ± 2.3 million (n = 5)). *Nosema* levels in bees fed 1.2 g of pollen (22.4 million spores/bee ± 1.3 million (n = 5)) did not differ significantly from those in bees fed other pollen amounts. Bees fed any amount of pollen had significantly higher *Nosema* levels than bees fed no pollen at all (2.1 million spores/bee ± 0.24 million (n = 5)).

**Fig 3:**
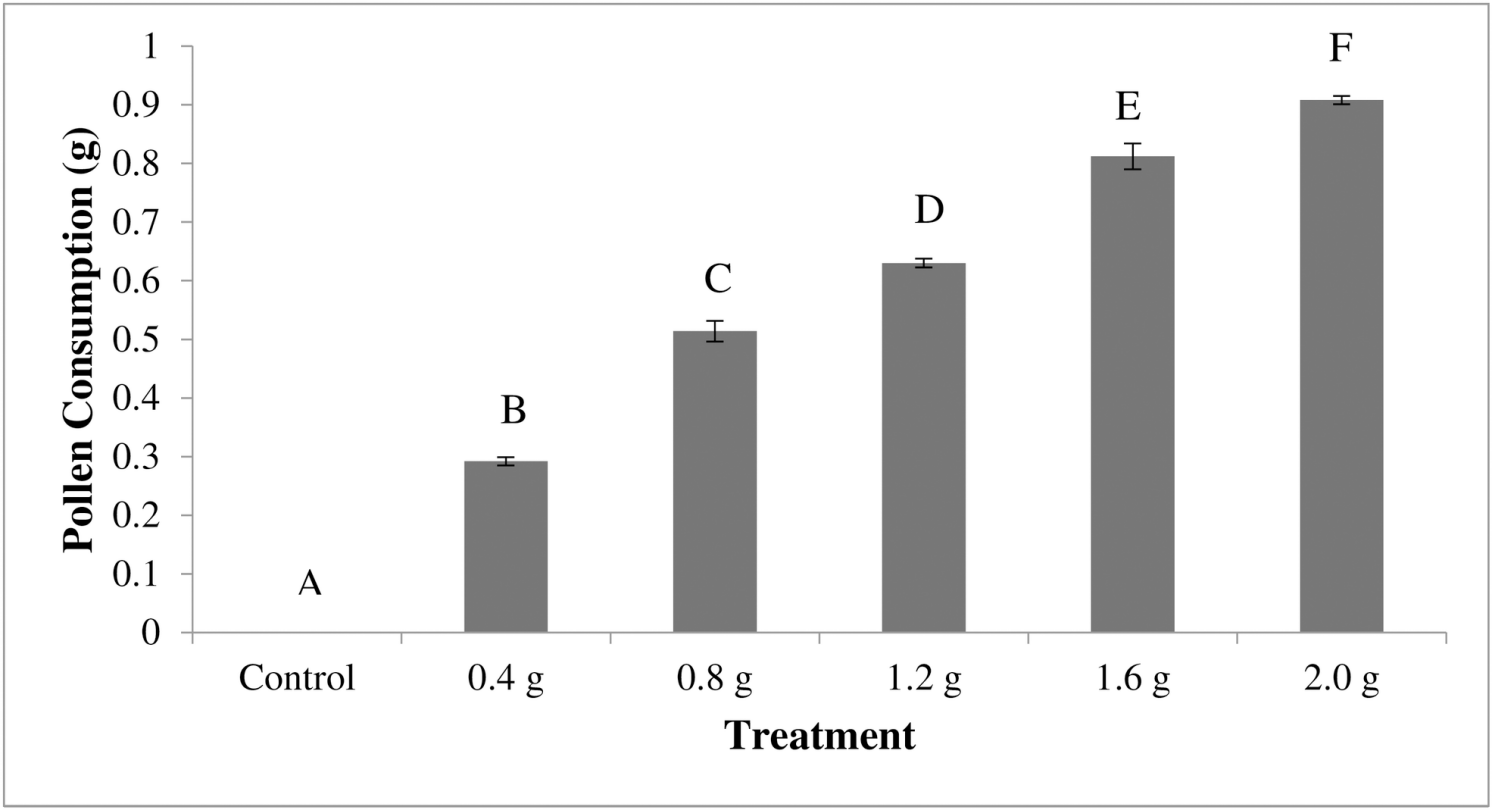
Consumption of pollen by bees across treatment groups. Data are the average amount of pollen consumed by the bees (in grams) with the error bars denoting standard error. N = 30 for all treatment groups. The treatment groups (x axis) represent the amount of pollen given to the cage of bees. Bees receiving more pollen consumed significantly more pollen across all treatment groups (ANOVA F_5, 24_ = 571.1, p<0.0001). Posthoc Tukey-HSD pairwise comparisons identified significance between (data with the same letter are not different at α ≤ 0.05).

While there were no significant differences between treatments in *Nosema* levels in bees fed ≥0.8 g of pollen, we did find significant differences in total consumption of pollen between each of the treatment groups (ANOVA F_5, 24_ = 571.1, p<0.0001, Fig 3). The groups of bees provided with 2.0 g, 1.6 g, 1.2 g, 0.8 g, and 0.4 g of pollen consumed a total of 0.90 g ± 0.02 g, 0.81 g ± 0.02 g, 0.63 g ± 0.01 g, 0.51 g ± 0.02 g, and 0.29 g ± 0.01 g pollen respectively. Mortality averaged 0.7 bees/cage across the six treatments and was not significantly different (ANOVA F_5,24_ = 2.4324, p = 0.0642).

### The impact of bee consumption of commercial diets on *Nosema* levels

**Fig 4:**
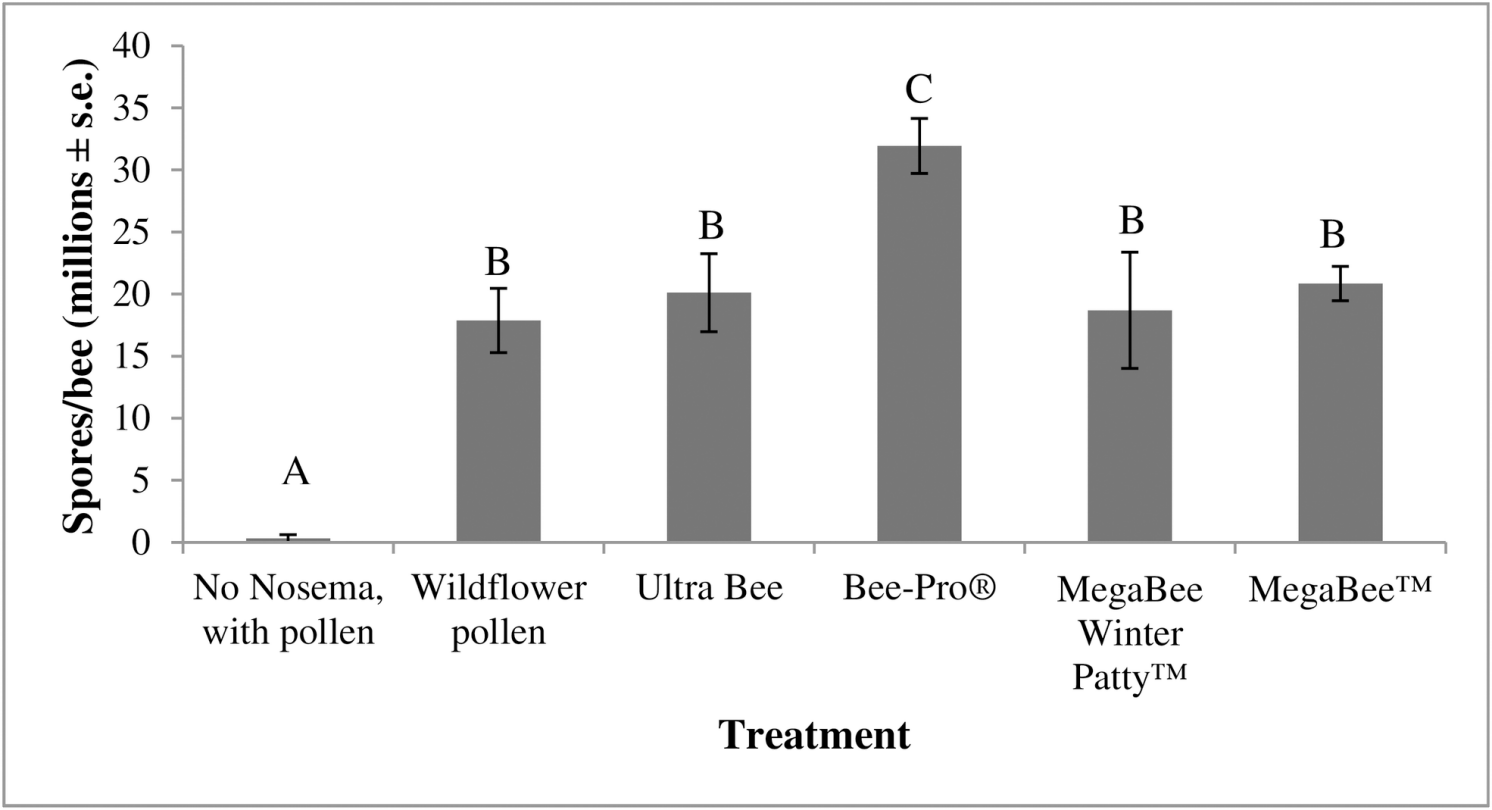
The impact of commercial pollen diet consumption by bees on *Nosema* levels in study 1 (fall 2013). Data are the average number of spores per bee (in millions) with the error bars denoting standard error. N = 56 for all treatment groups. The treatment groups represent the various commercial pollen substitute diets provided to bees. Treatment (type of diet) significantly affected *Nosema* levels in bees (ANOVA, F_5, 52_ = 15.9, p<0.0001). Posthoc Tukey-HSD pairwise comparisons identified significance between treatments (data with the same letter are not different at α ≤ 0.05).

There was a significant difference in study 1 between the level of *Nosema* in bees consuming the various commercial diets (ANOVA, F_5, 52_ = 15.9, p<0.0001, Fig 4). Bees fed Bee-Pro (31.9 million spores/bee ± 2.2 million) had significantly more *Nosema* than those fed wildflower pollen (17.9 million spores/bee ± 2.6 million), Ultra Bee (20.1 million spores/bee ± 3.1 million), MegaBee (18.7 million spores/bee ± 4.7 million), and MegaBee Winter Patty (20.8 million spores/bee ± 1.4 million).

**Fig 5:**
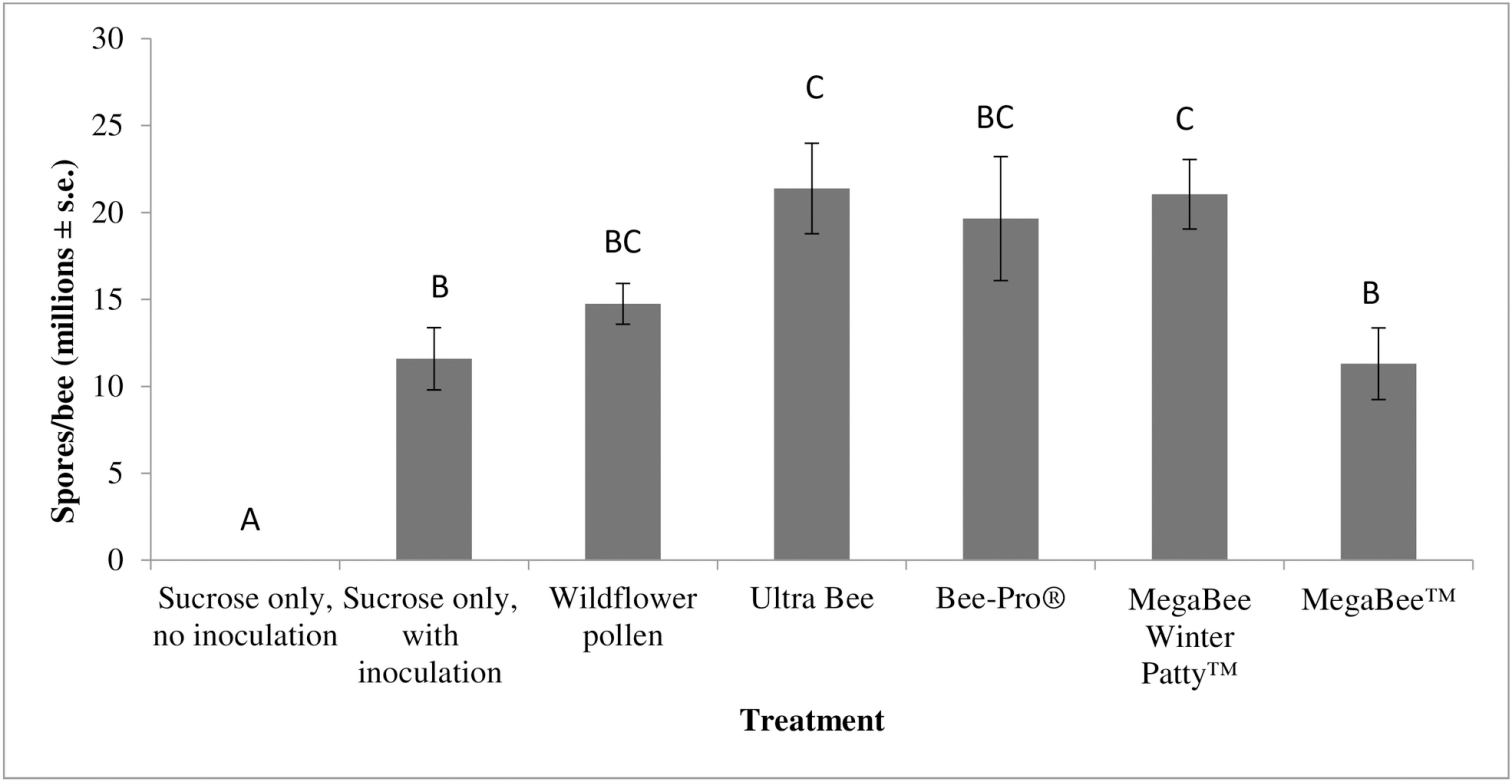
The impact of commercial pollen diet consumption by bees on *Nosema* levels in study 2 (spring 2014). Data are the average number of spores per bee (in millions) with the error bars denoting standard error. N = 70 for all treatment groups. The treatment groups represent the various commercial pollen substitute diets provided to bees. Treatment (type of diet) significantly affected *Nosema* levels in bees (ANOVA, F_5, 63_ = 33.6, p<0.0001). Posthoc Tukey-HSD pairwise comparisons identified significance between treatments (data with the same letter are not different at α ≤ 0.05).

Similar to study 1, there was a significant difference in study 2 between *Nosema* levels in bees fed the various commercial diets (ANOVA, F_5, 63_ = 33.6, p<0.0001, Fig 5). There were significantly higher *Nosema* levels in bees fed Ultra Bee (21.4 million spores/bee ± 2.5 million) and MegaBee Winter Patty (21.1 million spores/bee ± 1.9 million) than those fed only sucrose solution (11.6 million spores/bee ± 1.7 million) or MegaBee (11.3 million spores/bee ± 1.9 million). Bee-Pro- (19.6 million spores/bee ± 3.4 million) and wildflower-fed (14.8 million spores/bee ± 1.1 million) bees had intermediate *Nosema* levels that were not significantly different from those in bees fed the other commercial diets. Bees fed BeePro in study 1 and 2 had different *Nosema* levels from one another (ANOVA F_1,16_ = 8.10, p = 0.0212, Table 1).

**Table 1:** The seasonal differences between *Nosema* levels across treatment diets in study 1 (fall 2013) and study 2 (spring 2014).

Treatment	Year	*N*	Average number of *Nosema spores* ± s.e. (millions)	p-value
Wildflower	2013	9	17.87 ± 2.60	0.2723
	2014	10	14.75 ± 1.17	
Ultra Bee	2013	10	20.11 ± 3.14	0.7594
	2014	10	21.38 ± 2.61	
Bee-Pro	2013	9	31.93 ± 2.23	0.0212*
	2014	10	19.65 ± 3.57	
MegaBee	2013	8	18.69 ± 4.68	0.1396
	2014	10	11.3 ± 2.06	
MegaBee Winter Patty	2013	10	20.85 ± 1.38	0.9354
	2014	10	21.05 ± 2.00	

* denotes differences between years for spore numbers in bees for a given treatment at α ≤ 0.05.

The *Nosema* levels in uninoculated bees fed the various diets did not vary significantly and were negligible (F_5, 24_ = 0.9551, p = 0.4643, Table 2). Furthermore, bees consumed all diets equally (F_4, 20_ = 1.9294, p = 0.1448, Table 2). No single diet led to increased bee mortality (F_5,24_ = 1.3064, p = 0.2944, Table 2).

**Table 2:** Average levels of *Nosema* spores/bee, diet consumption per cage of bees, and bee mortality when fed the various diets in the absence of *Nosema* inoculum. *N* = 5 for all data.

Diet	Average number of *Nosema* spores/bee (millions ± s.e.)	Average amount of diet consumed per one cage of 15 bees (mg ± s.e.)	% bee mortality after 15 days (bees ± s.e.)
Sucrose only (control)	0.27 ± 0.10	N/A	2.2 ± 0.86
Wildflower	0	0.84 ± 0.05	0.4 ± 0.25
Ultra Bee	0.67 ± 0.43	0.88 ± 0.05	1.6 ± 0.60
Bee-Pro	0.18 ± 0.08	0.89 ± 0.02	1.2 ± 0.73
MegaBee	0.03 ± 0.03	0.85 ± 0.04	2.2 ± 0.49
MegaBee Winter Patty	0.83 ± 0.62	0.75 ± 0.03	1.2 ± 0.49

## Discussion

### The impact of inoculation route on *Nosema* levels in bees

We suspected that the level of *Nosema* in bees would be impacted by the route of inoculum delivery. Our data show that *Nosema* levels were much greater in bees that fed on inoculated pollen than in bees fed inoculated sucrose solution. Although *Nosema* spores are quite resilient to environmental conditions, it is possible that spores experience greater mortality in sucrose solution due to differences in pH between nearly neutral sucrose solution and the rather acidic environment of the honey bee midgut [41]. Others have demonstrated a measurable difference in *Nosema* polar tube anatomy based upon environmental moisture content [42] and this may impact the virulence of the spore once it is ingested by the bee. While using sucrose solution to inoculate bees with *Nosema* is the “standard” [38], our data suggest that the method used for *Nosema* inoculation in honey bee bioassays may impact the resulting findings and conclusions drastically.

### The impact of pollen consumption on *Nosema* levels in bees

Pollen is critical to the nutritional needs of the colony [43]. Our data suggest that *Nosema* does not replicate well in bees deprived of pollen. Inoculated bees provided at least 0.4 g of pollen per cage of 15 bees had over ten times higher *Nosema* levels than inoculated bees not receiving pollen, suggesting that bee nutrient intake is important to *Nosema* reproduction. Despite this, we did not find a positive correlation between *Nosema* levels and the amount of diet consumed by bees. Instead, our data suggest that any amount of pollen consumed by bees over a 0.8 g threshold produces statistically similar *Nosema* levels in bees.

Pollen consumption, even minute amounts, appears to contribute significantly to *Nosema* levels in bees. This finding is in agreement with similar studies in which the influence of diet on *Nosema* infection was evaluated [39]. Previous investigators showed that bees fed a diet containing pollen had six times more *Nosema* spores than bees fed a diet containing no pollen [39,44]. The researchers speculated that *Nosema* flourishes in a bee’s midgut when the bee is fed a more nutritious diet [39,44]. Alternatively it has been hypothesized that pollen consumption by bees could increase *Nosema* levels by increasing the surface area of the midgut, thus increasing the bee’s susceptibility to infection [39]. Since we did not see a linear increase in *Nosema* levels in bees fed more than 0.8g of pollen, we suspect that the level of *Nosema* in bees is dependent upon the nutritional content of the pollen or pollen substitute diet consumed by the bees and not an increase in the midgut internal surface area that results from bee consumption of diet.

### The impact of bee consumption of commercial diets on *Nosema* levels

Companies frequently claim multiple benefits associated with feeding bees pollen substitute/supplement diets. These claims include greater brood rearing, increased hygienic behavior, increased queen acceptance, increased resistance to colony stressors (including pathogens, parasites, and pesticides), and increased pollination and honey production resulting from use of a particular diet. As a result of these claims, we determined how the consumption of four commercial pollen substitute diets widely available in the U.S. impacts the number of *Nosema* spores in bees. While we confirmed that *Nosema* infection likely is not derived from the diets themselves, bee consumption of the different diets did produce varying levels of *Nosema* in inoculated bees.

It is unclear, however, what components in the pollen substitute diets are contributing to an increase in *Nosema* levels in bees. We know from past studies [39,44] that protein can increase the level of *Nosema* in bees, but the protein level in the commercial diets we tested did not correlate with the *Nosema* levels seen in the bees. Diets that had very low amounts of protein (i.e. MegaBee Winter Patty at 3% crude protein) had comparable levels of *Nosema* infection to those of diets containing high levels of protein (i.e. Ultra Bee at 60% crude protein). Our data suggest that *Nosema* infection may be dependent on other dietary factors. Furthermore, it is unknown how seasonal patterns are contributing to *Nosema* infection. We found differences between the contribution of BeePro to *Nosema* levels in bees in the fall and spring seasons despite inoculating bees with the same number of spores and providing the same amount of diet both seasons. *Nosema* levels were ~50% higher in bees fed the Bee Pro treatment in the fall than in bees fed the Bee Pro treatment in spring; however, we did not observe the same significant increase in *Nosema* levels across the other protein diets. Furthermore, there were no significant differences in the *Nosema* levels produced in bees eating the commercial diets in the spring when compared with bees fed wildflower pollen. Further investigation is needed to understand how the differences in the nutritional needs and physiology throughout the seasons of the year can impact *Nosema* and other pathogen infections.

The consumption of even small quantities of pollen is positively correlated with increased levels of *Nosema*. Additionally, there is a relationship between some commercial diets and *Nosema* levels that warrants further investigation. Our data suggest that supplemental pollen diet feedings may increase the level of *Nosema* infection in the colony. However, increases in *Nosema* infection have not been predictive of colony mortality. Furthermore, there currently are no thresholds for *Nosema* infection that would indicate a reduction in colony health when thresholds are reached. Beekeepers commonly feed their colonies throughout the year to prevent malnutrition. While pollen consumption may lead to higher *Nosema* reproduction within honey bees, the negative cost of inadequate nutrition that would result from withholding pollen and pollen substitutes (such as reduced brood production, immunocompetence, and foraging efficacy) to safeguard against *Nosema* may preclude modifying current management practices. It is unknown if we would observe the same impact of commercial pollen substitute diets on *Nosema* levels at the field level. Thus, field level studies would be integral to furthering our understanding on how to promote colony strength while simultaneously protecting honey bees from increases in parasite and pathogen infections.

## Supporting Information

S1 Fig Bioassay cage with adult honey bees. (TIF)
