# Supplementary figures and images for: Characterizing the Impact of Commercial Pollen Substitute Diets on the Level of Nosema spp. in Honey Bees (Apis mellifera L.)
Source: PLoS One. 2015 Jul 30;10(7):e0132014. doi: 10.1371/journal.pone.0132014 (PMC4520664; doi:10.1371/journal.pone.0132014)

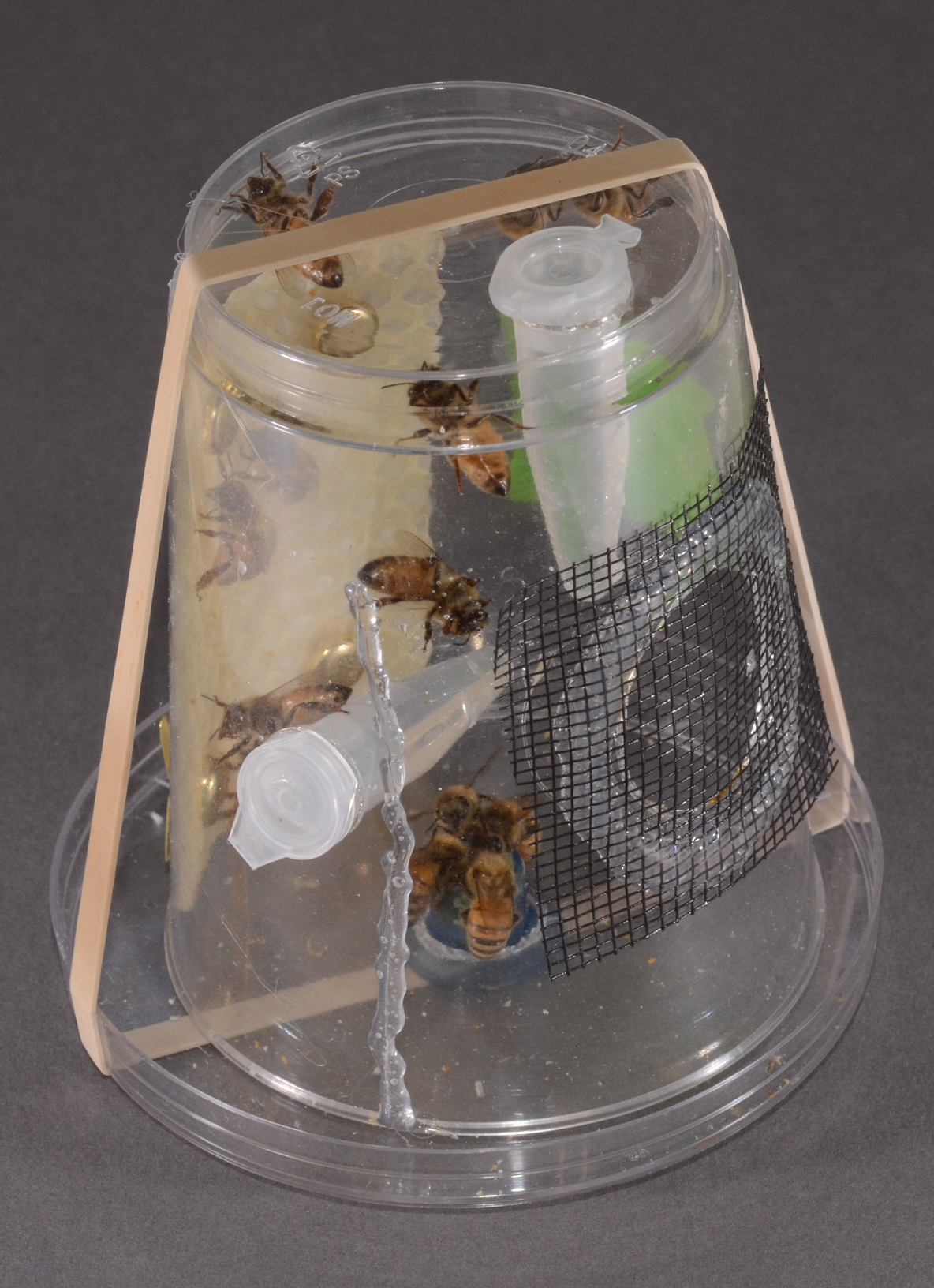

Supplement: S1 Fig — (TIF) [file pone.0132014.s001.tif]
